# Waste Baijiu Distillers’ Grain-Derived Porous Biochar: A Promising Material for Bisphenol AF Removal from Water Through Both Adsorption and Advanced Oxidation Process

**DOI:** 10.3390/molecules31101713

**Published:** 2026-05-18

**Authors:** Yi Xie, Jiali Yu, Yilong Li, Yongkui Zhang, Qulai Tang, Fangxiang Li, Yabo Wang, Bi Chen

**Affiliations:** 1 School of Brewing Engineering, Moutai Institute, Renhuai 564507, China; xieyi@mtxy.edu.cn (Y.X.); tangqulai07@foxmail.com (Q.T.); lifangxiang@mtxy.edu.cn (F.L.); 2 Kweichow Moutai Distillery Co., Ltd., Maotai Town, Zunyi 564501, China; yujl666999@163.com; 3 School of Chemical Engineering, Sichuan University, Chengdu 610065, China; liyilong2000@stu.scu.edu.cn (Y.L.); zhangyongkui@scu.edu.cn (Y.Z.)

**Keywords:** waste biomass, biochar, advanced oxidation process, bisphenol AF degradation, ecotoxicity

## Abstract

In recent years, accelerated industrialization has made water pollution a major challenge, bisphenol pollutants being one of the most typical examples. Advanced oxidation processes (AOPs) based on peroxymonosulfate (PMS) activation have been applied in environmental remediation due to their broad applicability and high pollutant removal efficiency. The key to AOPs lies in developing low-cost, highly active catalysts. This study utilized waste biomass of baijiu distillers’ grains (DSGs) as precursor to prepare biochar materials for bisphenol pollutant removal. Through high-temperature pyrolysis at 900 °C for 2 h in the presence of NaCl and KCl as activator, biochar-based materials (BC-x) were prepared, which possessed advantageous features of large specific surface area and high nitrogen doping content. When applied for typical bisphenol pollutant removal, the selected BC-900 biochar exhibited almost 100% bisphenol AF (BPAF) removal efficiency after a 30 min adsorption and following a 5 min PMS activation process under reaction conditions of 200 mg L^−1^ of BC-900, 200 mg L^−1^ of PMS, and 20 mg L^−1^ of BPAF. Reactive species of sulfate radicals (SO_4_⦁^−^), hydroxyl radicals (⦁OH) and singlet oxygen (^1^O_2_) were responsible for BPAF degradation, among which ^1^O_2_ played the major role. Further toxicity prediction of the BPAF degradation intermediate products implied the low ecological risk of the constructed BC-900/PMS catalytic system for BPAF removal. The findings in this study may provide useful guidance for waste biomass conversion and organic contamination remediation in water.

## 1. Introduction

In recent years, the global market has seen a continuous increase in demand for grain-based ethanol production. Concurrently, the output of its by-product, distillers’ grains (DSGs), has also risen. In 2019, the U.S. produced over 22 million tons of DSGs [1]. China is likewise another major producer of DSGs, considering the large quantity of baijiu production. During the baijiu fermentation process, after distillation, the residual grain components are mixed with rice hulls to form DSGs. Data indicates that China’s baijiu industry generates more than 100 million tons of distillers’ grain waste annually [2]. Due to the limitations and inefficiencies of traditional distillation techniques, proteins and fibers remain in the DSGs, making DSGs a typical waste biomass. Conventional organic solid-waste disposal methods may pose environmental risks, as landfills produce not only foul odors and greenhouse gases but also release pathogenic microorganisms and heavy metals from fermentation processes. These pollutants can contaminate soil and pose potential threats to agriculture and food safety [3]. Therefore, proper disposal of fermentation waste has become a research focus. Considering the high carbon element content, conversion of DSGs to biochar may be a feasible strategy.

Biochar is a low-cost and green carbon material with abundant precursors, adjustable pore structure, partially graphitized carbon framework, various surface functional groups and precursor-induced foreign element doping features. These advantageous material properties make biochar suitable for applications for pollutant removal via adsorption and catalytic processes. For example, biochar can adsorb pollutants through surface complexation, π-π interaction, hydrogen bonding, hydrophobic interaction, etc. [4,5]. Wang et al. successfully converted corn stover biomass into multilayered and porous carbon sheets via carbonization [6]. Heavy metal ions, metal oxyanions, and aromatic organic contaminates in water or soil environments could be accumulated by biochar materials via adsorption [7,8,9]. Biochar can also function as a catalyst for organic pollutant degradation through advanced oxidation processes (AOPs). For example, defect sites, graphitized carbon structure, N/P doping sites, and surface functional groups (e.g., C=O) are suggested to be reactive centers for peroxymonosulfate (PMS) and peroxydisulfate (PDS) activation [10,11,12]. The resultant reactive oxygen species (ROS, e.g., SO_4_⦁^−^, ⦁OH, ⦁O_2_^−^ and ^1^O_2_) attack organic pollutants and lead to their degradation into small molecules or even CO_2_ and H_2_O, achieving successful contamination remediation. Studies have shown that magnetic sludge biochar, pine needle biochar, and sludge biochar can activate persulfate (PS) to remove tetracycline hydrochloride [13], polychlorinated biphenyls [14], and 4-chlorophenol [15]. The defective structure in biochar and metal ions in the biochar produced from sludge can also promote the activation of persulfate. Zhu et al. prepared poplar biochar at 700 °C, activated PS, and reacted for 180 min to remove 97.8% clobeic acid (CA) [16]. Another study shows that fruit shell biochar prepared by slow pyrolysis is an efficient biosorbent, and the rice shell biochar/sulfite system can remove 99% of Cr (VI) in 120 min under a neutral environment [17,18]. TiO_2_-doped ferromagnetic composites synthesized from lignocellulosic orange peel biochar material by co-precipitation method can stably and efficiently degrade yellow-145 by Fenton reaction [19].

To obtain satisfactory pollutant removal efficiency, rational design of biochar structure is crucial. First, biomass precursor is the base of biochar structure tailoring. Various types of biomass originating from agricultural, forestry, fermentation, livestock and farming activities have been investigated for biochar production and environmental application [20,21,22,23]. Typically, biomass with high lignocellulose is regarded as a relatively pure precursor and suitable to produce biochar with fewer defects and a well-graphitized carbon framework. Mineral-element-containing biomass may result in biochar with more defects and foreign element doping. Biomass with high protein or microorganism content is a favorable precursor to obtain nitrogen-doped biochar. Second, the production method determines the physicochemical properties of biochar, e.g., yield, pore structure, surface functional groups, defects and graphitized structure [24,25,26]. Commonly, microwave carbonization, flash carbonization and roasting can produce biochar in a short time duration with high yield. The hydrothermal method facilitates the retention of abundant surface O-related functional groups. Oxygen-limited pyrolysis is feasible for the precise tailoring of biochar structure, which then becomes the preferred choice for biochar research. Third, pre-treatment and other reagent-assisted methods are proven to be efficient for the directional regulation of biochar structure [25,26,27]. In particular, molten-salt assistance is commonly accepted for pore structure tailoring of biochar. During the high-temperature dehydration and carbonization of biomass, the molten state of salt provides a special environment for the expansion of carbon framework, facilitating the formation of 2D carbon layer [28]. The salt component, especially potassium, can etch the carbon structure and create micropores [29,30]. Meanwhile, after pyrolysis, the crystallized salt particles will be removed by washing, leaving behind a porous structure. NaCl/KCl mixed salt, K_2_CO_3_, KOH, potassium citrate and potassium acetate are typical regents to assist in biochar structure tailoring [31,32,33].

This work aims to produce highly efficient biochar materials for organic pollutant removal from water, taking bisphenol AF as the model pollutant, due to its difficult degradation under natural conditions and its classical diphenyl ring and trifluoromethyl structure [34]. To meet the demands of advantageous structure features, waste biomass of baijiu DSGs with large annual production quantity and high nitrogen content was chosen as the biochar processor. NaCl/KCl mixed salt-assisted anoxic pyrolysis was applied for biochar production. Pyrolysis temperature was investigated as the key factor to tailor the structure of DSG-derived biochar. The ability of the resultant biochar materials for BPAF removal was evaluated through both adsorption and PMS activation-based AOP technology. A few environmental factors affecting AOP, e.g., catalyst and oxidant dosage, solution pH, and co-existing anions, were considered. Meanwhile, the AOP reaction mechanism was investigated. The ecotoxicity of original and AOP-treated BPAF solutions were preliminary predicated. This study may bring useful information for the treatment of both waste biomass and organic-contaminated water, as well as enrichment of the biochar material database.

## 2. Results and Discussion

### 2.1. Characterizations of DSG-Derived Biochar

**Figure 1:**
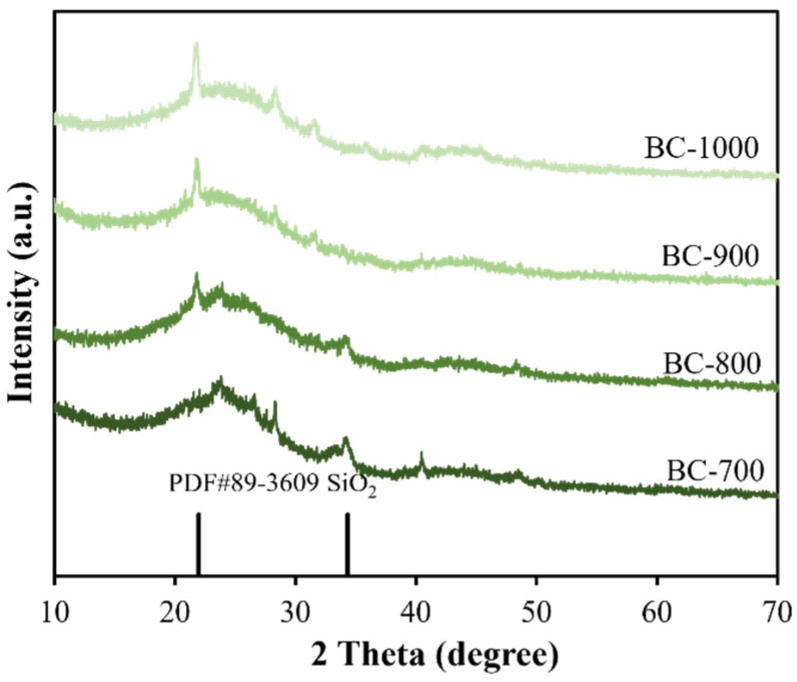
XRD patterns of DSG-derived biochar materials.

Figure 1 presents the XRD patterns of DSG-derived biochar materials. A broad and weak peak is observed at 2θ of ~23° for all samples, indicating the partially graphitized carbon framework in biochar [35]. The relatively sharp peaks located at 2θ of 21.9° and 33.6° are consistent with diffraction pattern of SiO_2_ (JCPDS card No. 89-3609), which originates from the silicon element present in DSGs. During the fermentation process, most lignocellulose in the grains is consumed and transformed by microorganisms, leaving inorganic elements in DSGs. As listed in Table 1, besides the C, H and O elements, considerable inorganic elements are detected, among which Si element shows a high content of 5.12 wt.%. Other small diffraction peaks shown in Figure 1 may be ascribed to the impurities brought by other inorganic elements in DSGs.

**Figure 2:**
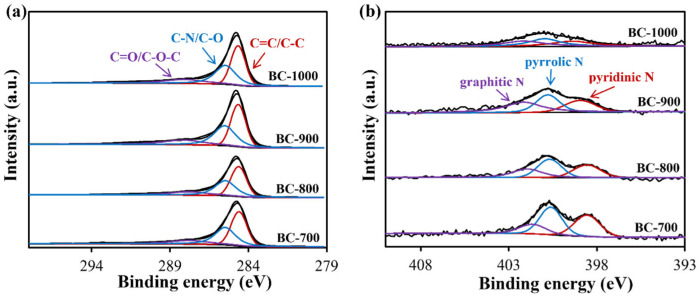
XPS C 1s (**a**) and N 1s (**b**) spectra of DSG-derived biochar materials.

The surface characteristics of DSG-derived biochar materials were analyzed by XPS. As depicted in Figure 2a, the high-resolution C 1 s spectrum of the prepared biochar indicates that carbon element primarily exists in the form of C=C/C-C chemical bonds. The rich N and O elements in DSGs lead to the observation of C-O and C=O bonds as well as a C-N bond. Although the pyrolysis temperature varied, the calculated carbon-related function groups on the biochar surface do not differ much. As listed in Table 2, C=C/C-C, C-O/C-N and C=O/C-O-C represent ~44%, ~38% and ~17%, respectively. However, the N-related species show a pyrolysis-temperature-dependent trend. As shown in Figure 2b and Table 2, the content of graphitic N continues to increase with increasing pyrolysis temperature and reaches a maximum of 40.46% at 900 °C. Further increasing pyrolysis temperature to 1000 °C leads to a slight decrease in graphitic N content. In contrast, the contents of both pyrrolic N and pyridinic N show a decreasing trend with the increase in pyrolysis temperature and reach the minimum at 900 °C. As commonly suggested in biochar-related AOP research [28], graphitic N is one kind of reactive center for PMS activation, which implies that BC-900 may be the advantageous sample for BPAF degradation through PMS activation. Furthermore, the comparison of the I_D_/I_G_ ratio of the Raman spectrum (Figure S1) shows that with the increase in pyrolysis temperature, the degree of defects in biochar first increases and then decreases, and reaches the maximum at 900 °C. The results of the infrared spectrum suggest that cellulose is very clear in the DSG sample, but there is basically no corresponding peak in the biochar sample of BC-900 (Figure S2).

**Figure 3:**
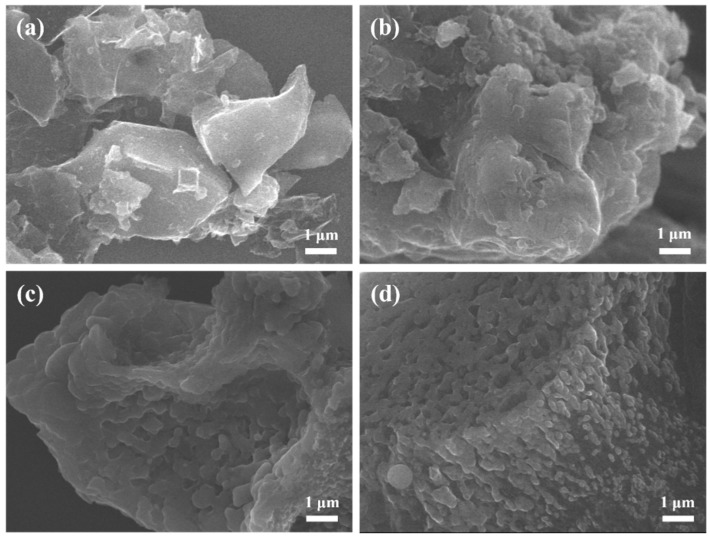
SEM images of DSG-derived biochar materials. (**a**) BC-700, (**b**) BC-800, (**c**) BC-900, (**d**) BC-1000.

**Figure 4:**
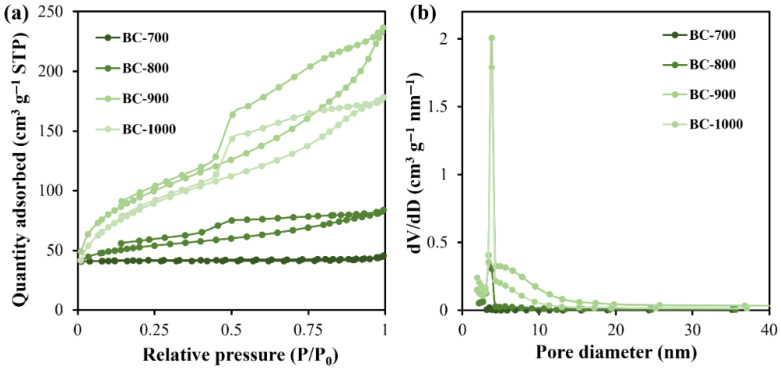
N_2_ adsorption–desorption isotherms (**a**) and pore size distribution curves (**b**) of DSG-derived biochar materials.

The morphological features of the biochar material were observed via FESEM and are displayed in Figure 3. An irregular and fragmented structure was observed with a particle size of a few micrometers (Figure 3a,b). It seems that pyrolysis temperature does not determine the overall morphology of biochar. However, the textural properties of biochar are highly dependent on pyrolysis temperature. With the increase in calcination temperature, the material appeared to have a more fragmented structure, which became tightly agglomerated biomass carbon blocks with irregular structure, and thin carbon sheets were formed on the surface of the material (Figure 3c,d). As shown in Figure 4a and Table 3, BC-700 biochar possesses a non-porous structure with an *S*_BET_ of only 3.6 m^2^ g^−1^, evidenced by the low N_2_ uptake in the N_2_ adsorption–desorption isotherm curve. With increasing pyrolysis temperature to 800 °C and 900 °C, a mesoporous structure is developed, as type IV adsorption–desorption isotherms with broad H3-type hysteresis loops are observed for BC-800 and BC-900 biochar (Figure 3a). Correspondingly, the *S*_BET_ values increase to 268.3 m^2^ g^−1^ and 524.1 m^2^ g^−1^ for BC-800 and BC-900, respectively. The pore size distribution curves (Figure 4b) and average pore diameter data (Table 3) also confirmed the mesoporous nature of BC-800 and BC-900 biochar. BC-900 samples have the highest pore volume distribution density in each pore size range, indicating that the material has more micropores or mesopores in a larger pore size range, which is conducive to the diffusion of BPAF or PMS molecules and the effective release of BPAF degradation products. At an elevated pyrolysis temperature of 1000 °C, the mesopores of biochar may collapse and eventually lead to the decreased *S*_BET_ and shrinkage of pore volume [36]. As the pyrolysis temperature increased from 700 °C to 1000 °C, the pore type distribution of the biochar exhibited a clear evolution. At 700 °C, all pore volumes were extremely low, indicating an underdeveloped porous structure. Upon raising the temperature to 800 °C, the micropore volume sharply increased to 0.10 cm^3^/g, becoming the dominant pore type. At 900 °C, the mesopore volume dramatically increased to 0.21 cm^3^/g, surpassing the micropore volume (0.15 cm^3^/g), resulting in a mesopore-dominated hierarchy with coexisting micro- and mesopores. Further increasing the temperature to 1000 °C led to a slight increase in micropore volume (0.20 cm^3^/g) but a sharp decrease in mesopore volume (0.09 cm^3^/g), suggesting that excessive temperature caused mesopore collapse or sintering, shifting the pore distribution back toward microporosity. It is noted from Table 3 that the BC-900 sample has the highest pore volume distribution density in each pore size range, indicating that the material has more micropores or mesopores in a larger pore size range, which is conducive to the diffusion of BPAF or PMS molecules and the effective release of BPAF degradation products.

### 2.2. Activities of DSG-Derived Biochar for BPAF Removal

**Figure 5:**
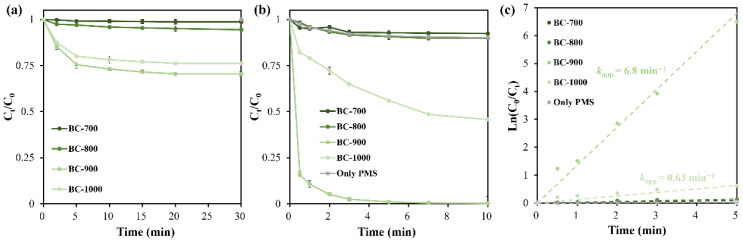
Removal of BPAF by DSG-derived biochar through adsorption (**a**) and PMS activation (**b**), and related data fitting by pseudo-first-order reaction kinetic model (**c**). Reaction conditions: [catalyst] = 200 mg L^−1^, [PMS] = 200 mg L^−1^, [BPAF]_initial_ = 20 mg L^−1^, pH unadjusted, 25 °C.

As one kind of carbon material with a porous nature and abundant surface functional groups, DSG-derived biochar shows both adsorption ability and catalytic degradation activity for BPAF removal from water. As shown in Figure 5a, DSG-derived biochar could remove BPAF pollutant by adsorption. In particular, under reaction conditions of 200 mg L^−1^ biochar dosage and initial BPAF concentration of 20 mg L^−1^, 30% of BPAF is quickly adsorbed by BC-900 in 30 min, corresponding to an adsorption capacity of 30 mg g^−1^. Other biochar materials show relatively low BPAF removal efficiencies, mainly ascribed to their insufficient specific surface area and pore volume.

When the oxidant of PMS was added into the equilibrized suspension containing biochar, the catalytic degradation process for BPAF was quickly initiated. Figure 5b shows that the four biochar materials are all capable of PMS activation to degrade BPAF, but their activities vary greatly. In contrast, PMS alone does not work for BPAF degradation, making the introduction of an efficient catalyst essential in this system. In detail, BC-700 can remove only 8% of BPAF in a 10 min catalytic process, corresponding a pseudo-first-order reaction rate constant (*k*_app._) of 0.09 min^−1^. Biochar obtained at 800 °C gives better PMS activation activity, enabling 10% of BPAF removal and *k*_app._ of 0.14 min^−1^. In particular, the BC-900 sample performs the best for BPAF degradation. During the first 5 min reaction process, almost all BPAF is degraded with a superior *k*_app._ of 6.8 min^−1^. The sample obtained at 1000 °C performs worse than that of BC-900. The BPAF removal efficiency is 54% and the corresponding *k*_app._ is 0.63 min^−1^. The well-developed pore structure with high *S*_BET_ and pore volume is crucial for heterogeneous catalysis; this is why BC-900 performs best in this study. However, it seems that pore structure is not the only determining parameter for PMS activation, since the normalized reaction rate constant by *S*_BET_ (*k*_app., norm.,_ min^−1^ m^−2^ g) followed the order of BC-700 (25 × 10^−3^) > BC-900 (12.97 × 10^−3^) > BC-1000 (1.43 × 10^−3^) > BC-800 (0.52 × 10^−3^). Surface functional groups should be another crucial factor affecting PMS activation and eventually BPAF degradation. It is reported that the surface C=O group plays the role of reactive center for PMS activation [37]. The N doping-enabled graphitized N species also function as the reactive site for PMS activation [28,38]. According to the XPS analysis shown in Table 2, the C=O content does not differ much among the biochar samples. But the content of graphitized N species shows the trend of BC-900 > BC-1000 > BC-800 > BC-700, which is consistent with the trend of *k*_app._ for biochar samples. Therefore, it is anticipated that graphitized N species play an important role in PMS activation in DSG-derived biochar material.

When comparing with reported data for BPAF degradation through PMS activation (Table 4), it is noticed that the DSG-derived biochar, especially the BC-900 sample, performs well with both high BPAF removal efficiency in a short time duration and fast reaction kinetics reflected by the high reaction rate constant (*k*_app._). Transition metal-based catalysts (e.g., Fe-, Mn-, Co-based catalysts listed in Table 4) are commonly found effective for PMS activation to degrade BPAF, reaching >90% removal efficiency in 30–120 min. However, metal leaching is always unavoidable due to the acidic environment created by PMS activation, which may cause secondary pollution. In contrast, metal-free catalysts (e.g., carbon materials) will not raise the concern of metal pollution. Meanwhile, they show even higher catalytic efficiency with faster reaction kinetics (Table 4). The main reason may be that both a radical mechanism and non-radical mechanism are present in the PMS activation process, contributing to BPAF degradation. Such a phenomenon is also observed in this study. In particular, the DSG-derived BC-900 sample demonstrates a high BPAF removal efficiency (>99%) with fast reaction kinetics (*k*_app._ = 6.8 min^−1^) under the reaction conditions comparable with reported works. It also should be mentioned that the selected BAPF concentration (20 mg L^−1^) is relatively high compared with reported work (Table 4). BC-900 still works well under the investigated BPAF concentration, implying its superior catalytic ability.

### 2.3. Effects of Typical Operation Parameters on BPAF Removal

**Figure 6:**
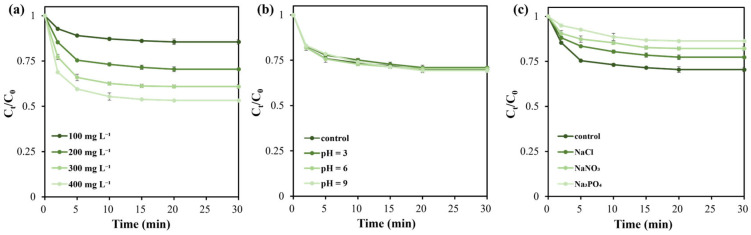
Effects of catalyst (BC-900) dosage (**a**), initial solution pH (**b**), and inorganic anions (**c**) on BPAF removal via adsorption process. Except for the investigated factor, other reaction conditions were kept as [catalyst] = 200 mg L^−1^, [BPAF]_initial_ = 20 mg L^−1^, [anion] = 10 mg L^−1^, pH unadjusted, 25 °C.

**Figure 7:**
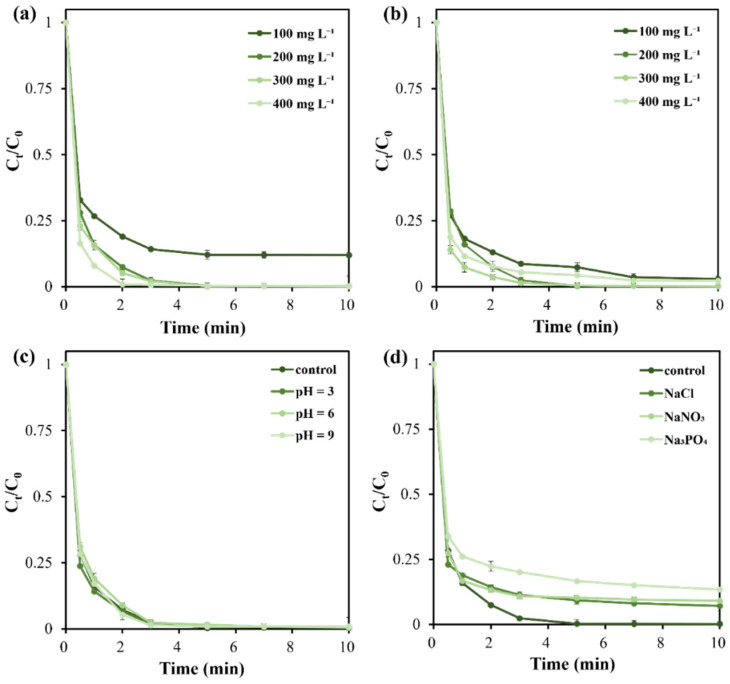
Effects of catalyst (BC-900) dosage (**a**), PMS dosage (**b**), initial solution pH (**c**), and inorganic anions (**d**) on BPAF degradation via PMS activation process. Except for the investigated factor, other reaction conditions were kept as [catalyst] = 200 mg L^−1^, [PMS] = 200 mg L^−1^, [BPAF]_initial_ = 20 mg L^−1^, [anion] = 10 mg L^−1^, pH unadjusted, 25 °C.

To investigate parameters affecting BPAF removal efficiency, a single-factor experimental design was employed to examine the influence of catalyst (BC-900) dosage, oxidant (PMS) dosage, initial solution pH, and inorganic anions on BPAF removal efficiency. The results are presented in Figure 6 and Figure 7.

In terms of adsorption efficiency, increasing the BC-900 biochar dosage from 100 mg L^−1^ to 200 mg L^−1^ elevated the BPAF removal efficiency from 15% to 30% (Figure 6a), since more adsorption sites are available. The degradation efficiency was also enhanced with the increase in BC-900 dosage (Figure 7a). At a dosage of 100 mg L^−1^, there is still 12% of BPAF left in the reaction solution after 10 min. Increasing BC-900 dosage to 200 mg L^−1^ could remove almost all BPAF after only a 5 min AOP process. Further increasing the catalyst concentration beyond 200 mg L^−1^ can continually improve the adsorption efficiency (Figure 6a), but it is not recommended since the catalytic process has limited enhancement (Figure 7a).

Increasing the oxidant dosage from 100 mg L^−1^ to 200 mg L^−1^ elevated the BPAF removal efficiency from 92% to 99% after 30 min adsorption and a 5 min catalytic process (Figure 7b). This occurs because adding more oxidants generates greater quantities of reactive oxygen species (ROS) that attack and mineralize pollutants. However, further increasing the oxidant dosage does not significantly enhance BPAF removal efficiency. Therefore, an oxidant dosage of 200 mg L^−1^ was selected for subsequent experiments.

As revealed in Figure 6b and Figure 7c, the initial pH of the BPAF solution had little effect on both the adsorption and catalytic processes. Under the investigated pH range of 3–9, the overall removal efficiency of BPAF can reach >99%, implying the wide pH adaptability of BC-900 biochar. Such a phenomenon was also found in other carbon-based catalysts for PMS activation [28,48], which could be ascribed to the stability of carbon catalysts and the non-radical mechanism involved in the AOP process.

Natural waterbodies often contain a large number of ions, which may interfere with the AOP process for BPAF removal. This study examined the effect of several typical ions (Cl^−^, NO_3_^−^, PO_4_^3−^) on the removal efficiency of BPAF. It is noticed that the adsorption process is first affected by the selected anions, showing a decreased BPAF removal efficiency (Figure 6c). The anions may affect the surface functional groups and compete for adsorption sites, leading to a decreased BPAF removal efficiency. Second, due to the quenching effect [51], the three anions further decrease the PMS activation activity and lead to the slowdown of reaction kinetics and the limitation of BPAF removal efficiency (Figure 7d). In particular, PO_4_^3−^ hinders BPAF degradation greatly, mainly due to its high affinity for the surface of carbon catalysts and coverage of active sites [28]. Although such a phenomenon is not expected and unfavorable, the total BPAF removal efficiency still reaches over 86%.

Considering the wide pH adaptability and relatively good anti-anion interference properties, the DSG-derived biochar, especially BC-900, possesses great potential for future application towards organic contaminate remediation in water.

### 2.4. Possible Catalytic Mechanism of BPAF Degradation Using BC-900 as Catalyst

**Figure 8:**
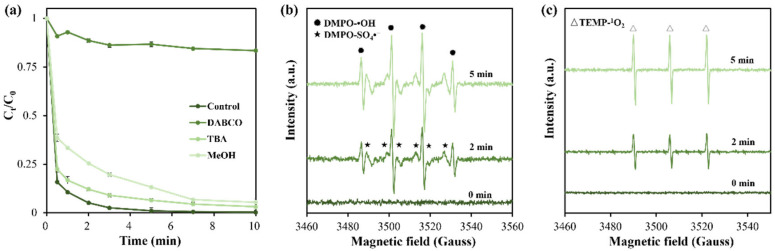
Reactive oxygen species quenching experimental results (**a**) and EPR spectra detected using DMPO (**b**) and TEMP (**c**) as trapping reagents.

Quenching experiments and EPR measurements were first conducted to identify the main reactive species responsible for BPAF degradation. As illustrated in Figure 8a, the presence of three quenchers in the reaction solution slows down reaction kinetics and decreases BPAF removal efficiency. In detail, TBA, the quencher of ⦁OH, hinders BPAF degradation to a removal efficiency of 93% after a 5 min catalytic process. MeOH, quencher of both SO_4_⦁^−^ and ⦁OH, further decreases BPAF removal efficiency to 86%. Particularly, when DABCO is added to the reaction suspension, a remarkable decrease in BAPF degradation efficiency to only 13% is observed, signifying the critical role of ^1^O_2_ in the catalytic process. Therefore, it is suggested that all three ROS contribute to BPAF degradation, while ^1^O_2_ plays the major role. Such a conclusion is further supported by EPR result. As depicted in Figure 7b, EPR signals representing DMPO-SO_4_⦁^−^ and DMPO-⦁OH complexes appear during in situ detection at 2 min reaction time, whose intensities enhance after 5 min reaction duration. Similarly, EPR signal of TEMP-^1^O_2_ adduct is also clearly observable and becomes stronger with prolonging reaction time. This indicates that oxidizing species of SO_4_⦁^−^, ⦁OH and ^1^O_2_ are continuously generated through the activation of PMS by BC-900 biochar.

Based on the above discussion, both radical pathways (ROS of SO_4_⦁^−^ and ⦁OH) and non-radical pathways (ROS of ^1^O_2_) are responsible for BPAF degradation. As a typical carbon catalyst with abundant surface functional groups, partially graphitized carbon structure and N doping feature, BC-900 provides efficient reactive sites for ROS generation through PMS activation. First, the C=O group is regarded as one kind of reactive center for PMS activation [37], which can interact with HSO_5_^−^ of PMS to form peroxide adduct. After a series of chain reactions, reactive species of ^1^O_2_ generate through the non-radical pathway. Second, N doping in biochar disrupts the pristine electronic and spin carbon configuration, resulting in the neighbor carbonyl carbon atoms with positive charge. Such a phenomenon is favorable for the anion of PMS to be attracted, which promotes the self-decomposition of PMS molecule and eventually formation of ^1^O_2_ via the non-radical process [28]. Third, the graphitized carbon framework and N doping-created graphitized N species can provide abundant free flowing of electrons and unpaired electrons, facilitating PMS activation to generate SO_4_⦁^−^ and ⦁OH radicals [52,53]. Additionally, N doping also leads to the formation of pyridinic N in biochar, which can act as a Lewis basic reaction site, contributing to the generation of SO_4_⦁^−^ and ⦁OH radicals [28]. Overall, the advantageous structure features of DSG-derived biochar, especially partially graphitized carbon framework and N doping, are synergistically responsible for BPAF degradation through PMS activation.

### 2.5. Reusability of BC-900 Catalyst

**Figure 9:**
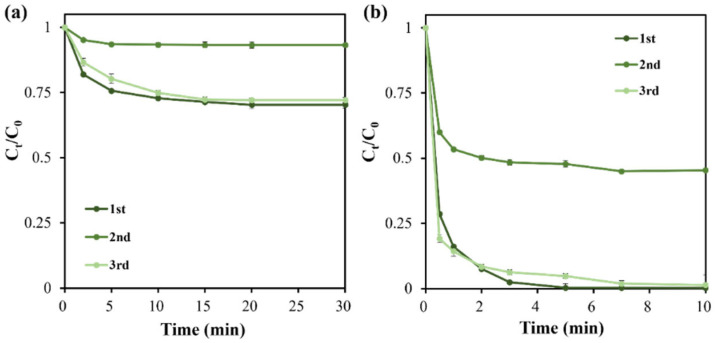
Reusability of BC-900 biochar for BPAF removal. (**a**) Adsorption process; (**b**) catalytic process.

The reusability of catalysts is a key indicator for practical application. As shown in Figure 9, BC-900 biochar performs well in the first reaction run for BPAF removal. However, its activity declines a lot in the second run, with adsorption efficiency dropping from 31% to 7% and degradation efficiency decreasing from 99% to 54%. The overall BPAF removal efficiency is only 58% in the second run. The XRD pattern of spent catalyst (Figure S3) is basically the same as that of BC-900 (Figure 1), indicating that the main structure of BC-900 is not affected. As previously reported, organic degradation intermediates covering the surface of exhausted catalyst is the main reason for catalytic activity loss in recycling runs [54]. Both adsorption sites and catalytic centers would be covered by such intermediates, hindering the interaction of both PMS and BPAF molecules, leading to the unacceptable decline of BPAF removal efficiency. Therefore, a regeneration method of calcination under Ar atmosphere was conducted for the used BC-900 biochar, which was further evaluated in the third run for BPAF removal. Fortunately, both the adsorption ability and catalytic activity are restored for BC-900 biochar in the third run. Therefore, it is recommended that a proper regeneration procedure, e.g., re-calcination, should be included for the application of BC-900 biochar in future.

### 2.6. Analysis of BPAF Degradation Intermediate Products and Degradation Pathways

**Figure 10:**
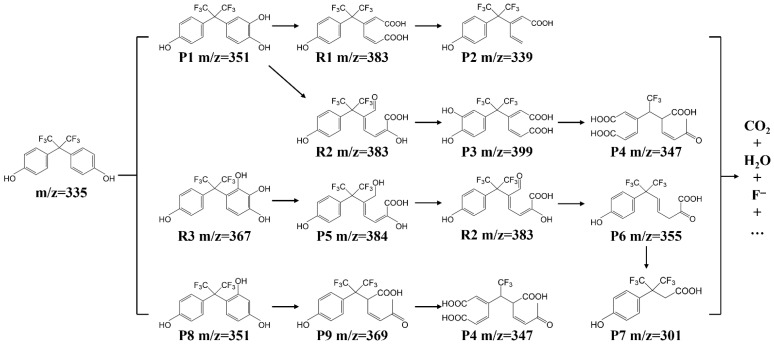
Proposed degradation pathways of BPAF in BC-900/PMS catalytic system. “P” and “R” represent detected and inferred intermediate products, respectively.

As indicated in the reusability evaluation (Figure 9), BPAF degradation intermediate products are generated in the BC-900/PMS catalytic system. Due to the difference in catalyst, reaction mechanism, and analysis method, the by-products of BPAF degradation varied in reported studies. For example, four by-products were detected in ferrate oxidation of BPAF, including hydroxylation products and C-C bond cleavage products [34]. In another study, a total of 10 by-products were reported through the bisulfite-activated persulfate oxidation of bisphenol AF [55]. Ortho-cleavage, metal-cleavage, ring-cleavage and C-C bond cleavage can occur in BPAF molecules and by-products through ROS attack, which are further oxidized into small molecules and eventually mineralized into CO_2_, H_2_O and F^−^. In this study, after LC-MS identification, a total of nine intermediate products were detected, including the ring-opening products, hydroxylated products, de-fluorinated products, etc. Based on the detected intermediate products and previously reported results, a proposed degradation pathway for BPAF is illustrated in Figure 10 [34,55,56]. Here, P denotes intermediate products detected by LC-MS, while R represents inferred intermediate products. Initially, the aromatic carbon atom in BPAF may be attacked by ⦁OH to generate hydroxylated products. Previous studies indicate that the ortho and para positions of the hydroxyl group in BPAF exhibit high electron density and are susceptible to oxidant attack [40]. The mono-hydroxylated products P1 and P8 are generated, which further undergo oxidative cleavage to form ring-opening products (R1, R2, P2). Then, decarboxylation reaction occurs to produce carboxylic acid compounds (P2, P4). Finally, they are mineralized into CO_2_, H_2_O, and inorganic F^−^ ions. Concurrently, P1 may undergo further hydroxylation under ⦁OH attack to form a dihydroxy product (R3). However, it is not detected in this study. This instability likely arises from the presence of two or more adjacent hydroxyl groups, rendering the polyhydroxy product structurally unstable. Consequently, these products readily oxidize to quinone-oxy intermediate P5 via ring opening. Subsequently, the -CH_2_OH group on P5 undergoes direct oxidation to form R2. The final reaction converts P6 to carboxylic acid compound P7, which is then mineralized into CO_2_, H_2_O, and inorganic F^−^ ions.

### 2.7. Preliminary Ecotoxicity Analysis of BPAF Degradation Products

**Figure 11:**
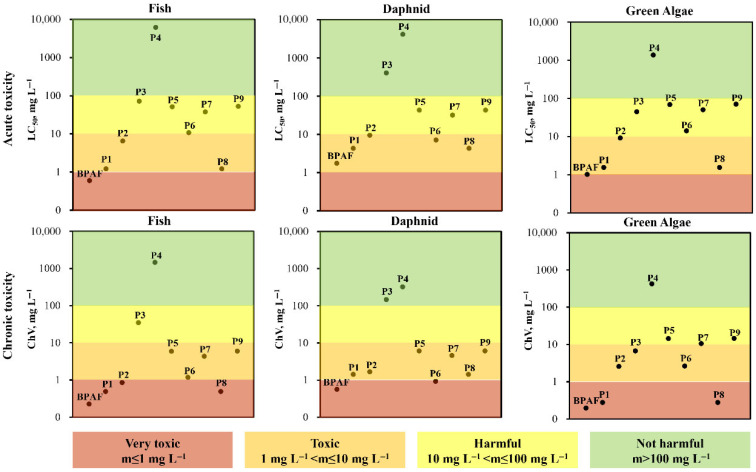
The predicted ecotoxicity of BPAF and its degradation intermediate products obtained from ECOSAR program. “m” represents LC_50_ or ChV.

Although the BC-900/PMS catalytic system can almost completely remove BPAF (Figure 5), the intermediate products generated during BPAF degradation may cause secondary environmental pollution. To assess the ecotoxicity of the intermediate products generated in the BC-900/PMS catalytic system during BPAF treatment, ecotoxicity predictions were made using the ECOSAR program (v2.2), taking aquatic organisms of fish, daphnid, and green algae as models. Based on the median lethal concentration (LC_50_, mg L^−1^) and chronic value (ChV, mg L^−1^) as evaluation criteria—where lower concentrations indicate higher toxicity—the results are categorized into four levels: no hazard, hazardous, moderately toxic, and highly toxic. As shown in Figure 11, in the presence of BPAF, the LC_50_ for fish is low, indicating that BPAF exhibits “high toxicity” to fish. Similarly, BPAF is moderately toxic to daphnid and green algae in terms of acute toxicity. As for chronic toxicity, BPAF is labeled as highly toxic to all the three aquatic organisms. In contrast, most of the BPAF degradation intermediates show significantly reduced acute and chronic toxicity. Some intermediate products, such as P3 and P4, can even be considered non-hazardous in most cases. Compared with BPAF, the acute and chronic toxicity of all intermediates to the three model organisms decreased in varying degrees. Previous studies also confirmed that most BPAF degradation by-products showed decreased toxicity towards model aquatic organisms, implying that ring opening, C-C bond cleavage and hydroxylation of BPAF could reduce the original toxicity of BPAF [48,57]. Therefore, although various intermediate products exist in the BC-900/PMS-treated BPAF solution, the ecotoxicity is suggested to be remarkably reduced.

## 3. Materials and Methods

### 3.1. Materials and Reagents

The distillers’ grains mainly from sorghum fermentation used in this study were provided by a local baijiu producer (Zunyi, China). The BPAF used in the study was of analytical grade and supplied by Aladdin (Riverside, CA, USA). Methanol was obtained from Fisher (Waltham, MA, USA) and supplied as HPLC grade. Other reagents of analytical grade were supplied by Chengdu Kelong Chemical Reagent Company (Chengdu, China).

### 3.2. Preparation of Biochar from DSGs

The as-received DSGs had a moisture content of ~50 wt.%, and were dried in a 60 °C oven for 12 h. The pre-dried DSGs were crushed and passed through a 35-mesh sieve. Then 2 g of sieved DSGs, 6 g of KCl and 6 g of NaCl were well mixed as the biochar precursor. The pyrolysis conditions included argon gas flow of 30 mL min^−1^, ramping rate of 5 °C min^−1^, holding temperature of 900 °C and holding time of 2 h. The soluble impurities and salt mixture in the pyrolyzed product were removed by washing with de-ionized water. After drying in a 60 °C oven for 12 h, biochar samples were obtained for further studies, which were denoted as BC-*x*. For example, BC-700 was the biochar sample obtained at pyrolysis temperature of 700 °C.

### 3.3. Characterization Techniques

The material properties were characterized by a powder X-ray diffractometer (XRD, Rigaku Ultima IV, Rigaku Corporation, Osaka, Japan), X-ray photoelectron spectroscopy (XPS, Thermo Fisher K-ALPHA, Thermo Fisher Scientific Inc., Waltham, MA, USA), field emission scanning electron microscope (FESEM, FEI Apreo S, Thermo Fisher Scientific Inc., Waltham, MA, USA), N_2_ physical adsorption instrument (Micromeritics^®^ ASAP 2460, Malvern Panalytical company, Malvern, UK), Fourier transform infrared spectrometer (FTIR, Spectrum Two, Perkin Elmer, Shelton, CT, USA), Raman spectrometer (Raman, DXR, Thermo Scientific, Waltham, MA, USA) and X-ray fluorescence spectroscopy (XRF, Rigaku Supermini 200, Rigaku Corporation, Osaka, Japan). Electron paramagnetic resonance (EPR) spectra were measured to identify the reaction oxygen species (ROS) generated in AOPs. The BPAF degradation intermediates were identified by liquid chromatography–mass spectroscopy (LC-MS).

### 3.4. Activity Evaluation of As-Obtained Biochar for BPAF Removal

In a typical run, 200 mg L^−1^ biochar sample was added into 200 mL BPAF aqueous solution (20 mg L^−1^) under constant stirring. The adsorption process lasted for 30 min to reach adsorption–desorption equilibrium. Then, 200 mg L^−1^ of PMS was added into the suspension to initiate AOP reaction. At a pre-designated time point, 0.5 mL of the suspension was withdrawn and mixed with equal volume of methanol immediately to end the AOP reaction. After filtering with a 0.22 μm membrane filter, the BPAF concentration in the filtrate was quantified via high-performance liquid chromatography (HPLC, Shimadzu Essentia LC-16, Shimadzu Corporation, Kyroto Japan). The detection wavelength for BPAF was 280 nm. The selected HPLC column was a SHIMSEN Ankylo C18 column (250 mm × 4.6 mm, 5 μm). The injection volume was 20 μL.

The radical quenching experiments were conducted with methanol (MeOH), tert-butyl alcohol (TBA) and 1,4-diazabicyclo[2.2.2]octane (DABCO) as the quenchers of both sulfate radicals (SO_4_⦁^−^) and hydroxyl radicals (⦁OH), ⦁OH only, and singlet oxygen (^1^O_2_), respectively.

## 4. Conclusions

This study employed waste biomass of baijiu distillers’ grains (DSGs) as feedstock to produce biochar materials for BPAF-contaminated water remediation through adsorption and PMS activation-based AOPs. The fermentation residues in DSGs, e.g., proteins and microorganisms, enabled the production of biochar with high N doping content, while the molten-salt-assisted pyrolysis improved the textural structure of biochar, such as high specific surface area and large pore volume. Together with the partially graphitized carbon framework, these advantageous structure features facilitated BPAF removal from water via both adsorption process and subsequent PMS activation procedures. Eventually, almost all BPAF could be removed in a short reaction time duration (e.g., 30 min adsorption and 5 min catalytic process). Further studies revealed that SO_4_⦁^−^, ⦁OH and ^1^O_2_ were responsible for BPAF degradation, while ^1^O_2_ played the dominant role. Although BPAF could not be totally mineralized into CO_2_ and H_2_O, the ecotoxicity of the residual degradation intermediate products was recognized as relatively low compared to that of the pristine BPAF. This study may provide useful guidance for waste biomass valorization and enrichment of the biochar database for organic pollutant remediation.

## Figures and Tables

**Table 1:** Elemental composition of DSGs measured by XRF.

Element	Si	K	Fe	Ca	P	S	Al	Cl	Mg
wt.%	5.12	3.73	1.78	1.73	1.57	1.11	0.82	0.68	0.38

**Table 2:** Contents of surface functional groups of DSG-derived biochar materials obtained from XPS.

Functional Group	BC-700	BC-800	BC-900	BC-1000
C=C/C-C *^a^*	44.63%	44.08%	43.58%	43.76%
C-O/C-N *^a^*	38.49%	38.46%	37.89%	39.03%
C=O/C-O-C *^a^*	16.88%	17.46%	18.53%	17.21%
graphitic N *^b^*	19.00%	29.70%	40.46%	33.22%
pyrrolic N *^b^*	43.75%	39.18%	33.34%	35.62%
pyridinic N *^b^*	37.25%	31.12%	26.20%	31.16%

*^a^* calculated from Figure 2a; *^b^* calculated from Figure 2b.

**Table 3:** Textural properties of DSG-derived biochar materials.

Material	*S*_BET_ (m^2^ g^−1^)	Total Pore Volume (cm^3^ g^−1^)	Micropore Volume (cm^3^ g^−1^)	Mesopore Volume (cm^3^ g^−1^)	Macropore Volume (cm^3^ g^−1^)	Average Pore Diameter (nm)
BC-700	3.6	0.008	0.001	0.004	0.003	8.12
BC-800	268.3	0.18	0.10	0.04	0.04	3.78
BC-900	524.1	0.46	0.15	0.21	0.10	4.61
BC-1000	440.6	0.35	0.20	0.09	0.06	3.75

**Table 4:** Comparison of reported catalysts for PMS activation towards BPAF degradation.

Catalyst	Reaction Conditions	BPAF Removal	*k*_app._ (min^−1^)	Reference
FeMoO_4_	[catalyst] = 0.2 g L^−1^ [PMS] = 4 mM [BPAF]initial = 5 mg L^−1^	95.2% in 60 min	0.049	[39]
Amino-modified MOFs	[catalyst] = 0.1 g L^−1^ [PMS] = 0.25 mM [BPAF]initial = 5 mg L^−1^	94.1% in 60 min	0.04	[40]
Mn_3_O_4_	[catalyst] = 0.5 g L^−1^ [PMS] = 4 mM [BPAF]initial = 5 mg L^−1^	90% in 90 min	0.024	[41]
BiFeO_3_	[catalyst] = 0.5 g L^−1^ [PMS] = 0.25 mM [BPAF]initial = 5 mg L^−1^	94.7% in 60 min	0.022	[42]
BiO_0.5_Cl_0.5_	[catalyst] =1 g L^−1^ [PMS] = 0.3 mM [BPAF]initial = 10 mg L^−1^	98.24% in 45 min	0.0959	[43]
Mn_0.2_Co_0.8_O_x_	[catalyst] = 0.4 g L^−1^ [PMS] = 0.2 mM [BPAF]initial = 5 mg L^−1^	97.7% in 40 min	0.074	[44]
NaBiO_3_	[catalyst] = 0.5 g L^−1^ [PMS] = 0.5 mM [BPAF]initial = 6.7 mg L^−1^	92.3% in 30 min	0.078	[45]
Manganese-containing minerals	[catalyst] = 1 g L^−1^ [PMS] = 0.4 mM [BPAF]initial = 3.3 mg L^−1^	83% in 120 min	0.0152	[46]
SB-BC-900	[catalyst] = 0.2 g L^−1^ [PMS] = 0.3 mM [BPAF]initial = 20 mg L^−1^	93.7% in 10 min	Not provided	[47]
N,P-co-doped carbon nanosheet	[catalyst] = 0.06 g L^−1^ [PMS] = 0.66 mM [BPAF]initial = 20 mg L^−1^	100% in 15 min	0.4115	[48]
N-doped carbon-based materials	[catalyst] = 0.3 g L^−1^ [PMS] = 1.3 mM [BPAF]initial = 20 mg L^−1^	100% in 30 min	0.1729	[49]
Heat/CoFe_2_O_4_@BC	[catalyst] = 0.1 g L^−1^ [PMS] = 1 mM [BPAF]initial = 20 mg L^−1^	87% in 30 min	0.0755	[50]
**BC-900**	**[catalyst] = 0.2 g L^−1^** **[PMS] = 1.3 mM** **[BPAF]initial = 20 mg L^−1^**	**99% in 5 min**	**6.8**	**This work**

## Data Availability

The original contributions presented in this study are included in the article.

## Supplementary Materials

The following supporting information can be downloaded at https://www.mdpi.com/article/10.3390/molecules31101713/s1. Figure S1: Raman spectra of DSGs-derived biochar samples; Figure S2: FTIR spectra of DSGs and DSGs-derived biochar samples; Figure S3: XRD of spent BC-900 sample.
