# Supplementary material for: Waste Baijiu Distillers’ Grain-Derived Porous Biochar: A Promising Material for Bisphenol AF Removal from Water Through Both Adsorption and Advanced Oxidation Process
Source: Molecules. 2026 May 18;31(10):1713. doi: 10.3390/molecules31101713 (PMC13210154; doi:10.3390/molecules31101713)
Supplement: Supplementary file 1 [file molecules-31-01713-s001.zip › molecules-4278333-supplementary.pdf]

Supplementary Materials for:

Waste Baijiu Distillers' Grains-derived Porous Biochar: A Promising Material for Bisphenol AF Removal from Water through both Adsorption and Advanced Oxidation Process

Yi Xie [1,+], Jiali Yu [2,+], Yilong Li [3], Yongkui Zhang [3], Qulai Tang [1], Fangxiang Li [1], Yabo Wang [3,*] and Bi Chen [2,*]

[1] School of Brewing Engineering, Moutai Institute, Renhuai 564507, China; xieyi@mtxy.edu.cn (Y.X.); tangqulai07@foxmail.com (Q.T.); lifangxiang@mtxy.edu.cn (F.L.)

[2] Kweichow Moutai Distillery Co., Ltd., Maotai Town, Zunyi 564501, China; yujl666999@163.com

[3] School of Chemical Engineering, Sichuan University, Chengdu 610065, China; liyilong2000@stu.scu.edu.cn (Y.L.); zhangyongkui@scu.edu.cn (Y.Z.)

* Correspondence: ybwang@scu.edu.cn (Y.W.); chenbi0703@163.com (B.C.)

† These authors contributed equally to this work.

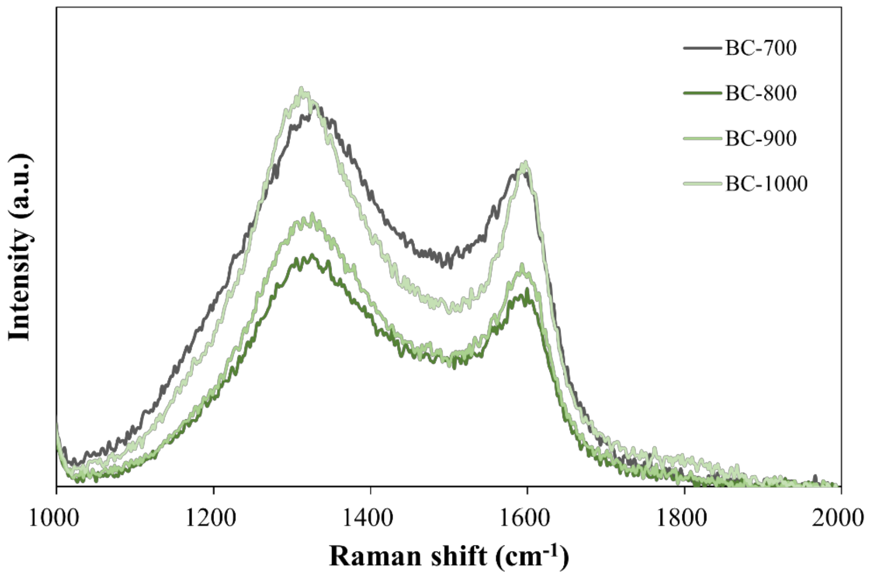

Figure S1. Raman spectra of DSGs-derived biochar samples

Figure S1 shows that the two distinct peaks locating at around 1325 cm^{-1} and 1592 cm^{-1} are indexed to D and G band. The I_D/I_G values of BC-700, 800, 900, 1000 are measured to be 1.19, 1.22, 1.23, 1.19, respectively. It is obvious that with the increase of pyrolysis temperature, the degree of defects first increases and then decreases, and reaches the maximum at 900 °C.

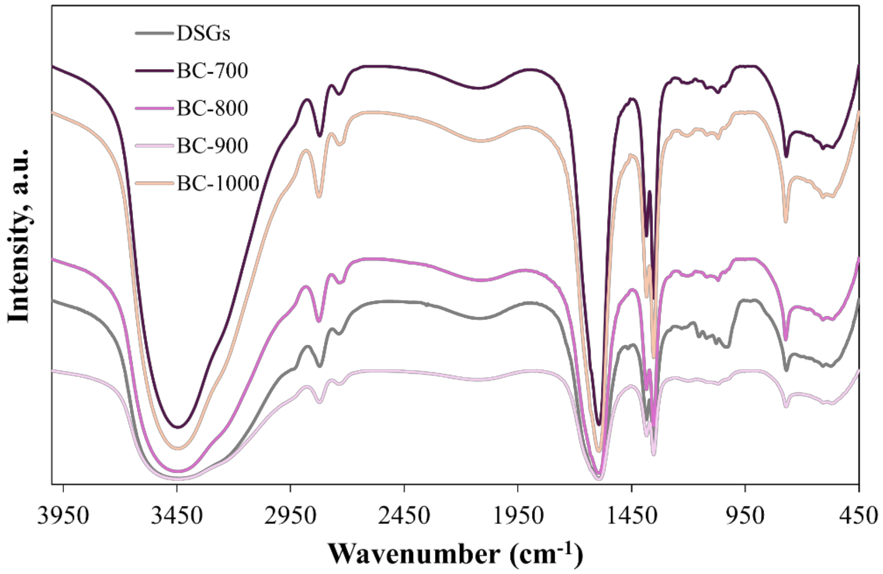

Figure S2. FTIR spectra of DSGs and DSGs-derived biochar samples

As shown in Figure S2, the most obvious difference in the spectrum is that the

1080 cm^{-1} and 1050 cm^{-1} double peak representing cellulose is very clear in DSG sample, but there is basically no corresponding peak in biochar sample of BC-900.

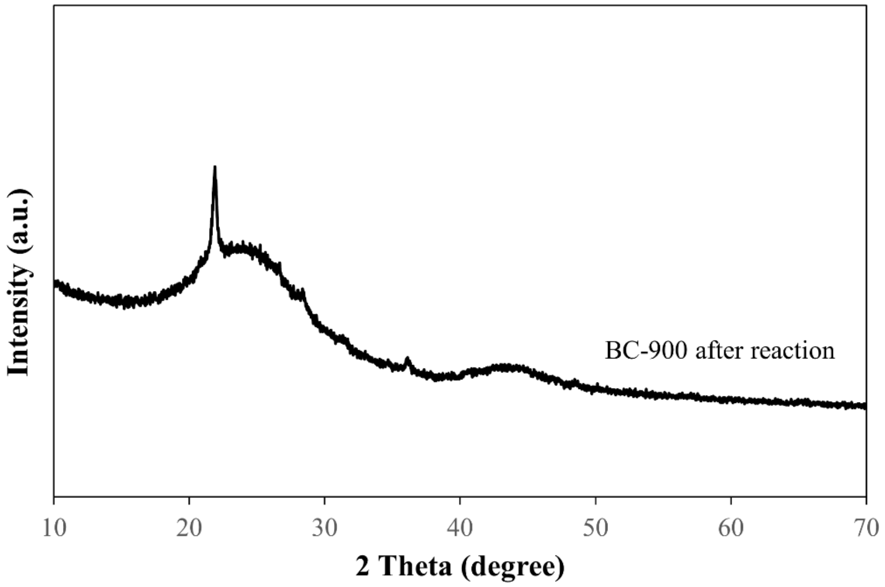

Figure S3. XRD of spent BC-900 sample

As shown in Figure S3, The XRD pattern of spent catalyst is basically the same as that of BC-900 (Fig. 1 in the main text), including the partially graphitized carbon framework in biochar (23°) and SiO_2 (21.9° and 33.6°).
